# Small-Pox and War

**Published:** 1917-01-13

**Authors:** 


### SMALL-POX AND WAR.

### What Has Happened and may Happen Again.

THE dread of small-pox, as a loathsome, disfiguring, and often fatal disease, likely periodically to become epidemic, has almost died out, and has affected popular opinion and conduct in this country but little since the outbreak of 1901-02. The possibility of a recrudescence of small-pox as a direct result of the present war, though popularly disregarded, has not failed to impress the sanitary and military authorities, who are fortunately fully alive to the need of maintaining in readiness the necessary machinery for dealing with it should it arise. The importance and danger of small-pox as a "war epidemic" are deserving of serious regard. In view of the wonderful improvement in sanitary conditions, the control of epidemic disease is a problem which has been in great measure solved. This very improvement has, however, in the case of small-pox, weakened the line of defence afforded by vaccination and strengthened the hands of those who have seized upon every opportunity to bring vaccination into disusage and even disrepute.

The Franco-Prussian War of 1870-71, the only great war in Western Europe within living memory, was followed not only by the spread of small-pox amongst combatants, but by the occurrence of the disease in epidemic form throughout Germany and, though with less severity, in all the neighbouring countries. Though Great Britain and Ireland were in great measure protected by their insular position and by the fact that they were not participants in the war, there was constant intercourse across the Channel, and infection was conveyed, by French refugees and by sailors, to our seaport towns, as well as to London and other centres of large population. The death-rate from small-pox in England, which had declined to about 1.0 per 10,000 inhabitants, increased to 10.1 in 1871 and 8.3 in 1872; London had a rate in 1871 of 24.2. The accompanying increase in more distant parts did not fully show itself until 1872, when the rate for Scotland reached 7.2 and that for Ireland 6.2.

On the continent of Europe, and especially in Germany, there raged an epidemic of small-pox the extent and virulence of which were unequalled by any other epidemic of the nineteenth century. A very full account of this is given in "Epidemics Resulting from Wars," * by Dr. Friedrich Prinzing, who has collated the facts in great detail. It may be stated at once that the German combatant forces suffered but little; the active forces were well protected by vaccination and re-vaccination; and, although 14,648 deaths in the army were attributed to disease, only 1.9 per cent. of these deaths were from small-pox; the immobile German troops (which were engaged in fortresses away from the fighting-line, and in guarding prisoners, etc.), consisting largely of reserves, were hurriedly called together and, being less efficiently protected by vaccination, suffered relatively more.* The French was not a re-vaccinated army; there had been great laxity, and many soldiers had never been vaccinated at all: it was amongst the French troops that small-pox spread, and by them infection was conveyed far and wide.

Amongst the civil populations freedom from recent serious epidemic outbreak, apathy, and the activity of opponents had caused vaccination to be widely neglected. In France one-third at least of the population was unvaccinated. Those who have been in the habit of belauding the perfection of German scientific organisation may be surprised that, whilst all recruits to the Prussian Army had been vaccinated since 1834, vaccination had been widely evaded by civilians throughout Germany, and in Prussia had not been compulsory. It was not until later, in 1874, that vaccination and re-vaccination were enforced by law throughout Germany. Before 1870 small-pox was not prevalent in Germany. The mortality in Prussia from 1868 to 1870 had been at the rate only of 1.8 per 10,000; in some States the rate was still lower. Germany may be said to have been almost free from small-pox in the summer of 1870. In France, on the other hand, the rate increased in 1869; in 1870 the disease was epidemic in several parts of the country, and it continued to spread.

From all parts of France small-pox was carried, with the mobilisation of troops, to the seat of war. Under war conditions infection spread readily amongst the French soldiers; of these large numbers were from time to time scattered as prisoners throughout Germany. The German troops were, as we have seen, but little susceptible. The case of the civil population was, however, different; small-pox everywhere broke out amongst the French prisoners, and from them the German guards and the civilians, alike insufficiently protected and taken unawares, were infected. Dissemination was easy, and was "conveyed from place to place, often considerable distances, by the moving population itself, not infrequently by marching troops and particularly by removal of prisoners from one place of detention to another. . . . The prisoners at Mayence, Coblenz, Wesel, Muiden, etc., became the foci from which the disease was transplanted into hitherto uninfected places." The stories from widely-scattered localities bear a striking resemblance one to another. We read of the arrival of prisoners, the occurrence of a case or two of small-pox amongst them, and the isolation of the patients. Then a laundress, an attendant, or a military guard carried home the infection, which spread with more or less activity according to local peculiarities. Not infrequently conveyance was attributed to the sale or distribution of blankets or clothes which had been

* *Epidemics Resulting from Wars.* By Dr. Friedrich Prinzing. (Carnegie Endowment for International Peace.) Oxford: Clarendon Press. 1916. This book was reviewed in THE HOSPITAL on October 14, 1916.

* A comparable instance occurred in a London workhouse in 1902. It was thought well to re-vaccinate the younger inmates first: as a consequence, a relatively large number of cases of small-pox arose amongst old people—amongst those whose protection was delayed!

used by the prisoners in the lazarets. An interesting and somewhat remarkable fact frequently noted was the slowness of dissemination: two or three months often elapsed before a local epidemic reached its height; and in outlying regions the greatest prevalence was delayed until 1872. The French prisoners in Germany were eventually vaccinated.

It may be of interest to refer briefly to one or two of the well-known places affected. Berlin had been but little troubled by small-pox since 1801, when 1,626 out of 176,700 inhabitants had succumbed. The "war epidemic" was unusually severe and virulent, 15 per cent. of the patients dying in the hospitals; whilst for 1871 the death-rate per 10,000 inhabitants reached 63.1. Hamburg, which had suffered somewhat severely (19.7 deaths per 10,000) in 1864, had but five deaths from small-pox in 1868. In the years 1870-72 the deaths numbered 4,053: vaccination was enforced in January 1872. In Cologne, whilst in the German garrison of 9,207 men there were but nineteen cases of the disease and one death, the mortality-rate for 1871 was 32.2 per 10,000 amongst the civil population. To Coblenz 15,011 French prisoners were taken; in one month (January) 571 cases occurred amongst them, and of these 111 were fatal; the usual outbreak occurred in the town and district.

The small-pox epidemic in Germany which followed directly upon the war of 1870-71 cost the country some 170,000 lives. In Prussia a mortality-rate of 1.8 per 10,000 (1868-70) became one of 25.6 (1871-72); by 1874 it had again fallen to 1.0. All parts of France also suffered severely. The existing epidemics were intensified and fostered as the result of the war. Vacher estimates the number of deaths caused by the disease in the two years 1870 and 1871 at not less than 100,000.

Fortunately, the conditions of this country to-day are in no way so favourable to the spread of small-pox as were those of France in 1870; nor are we endangered by the exigencies of war as were the Germans at that time. So good is our public health organisation, and so great is the reliance placed upon it, that a widespread neglect of vaccination has been permitted for many years past. It seems, at first sight, strange that now when vaccination is simple, cleanly, and safe, when the immunity obtainable by it has become in some degree explicable, and when analogous methods of prophylaxis are in common use, vaccination should have fallen into popular disfavour. The present position has only to a very small extent resulted from the propaganda of active anti-vaccinationists: these theorists may have encouraged and even cultivated the earlier variety of conscientious objector, but the objections to vaccination are mainly due to the lack of any obvious and pressing necessity for it. Faith in the efficacy of vaccination remains unshaken; vaccination or re-vaccination is accepted with but little demur by recruits for the Navy and Army, and readily by visitors to small-pox patients in hospitals; the protection afforded by it is sought with eagerness (even, it is said, by professed anti-vaccinationists of the "rabid" school) in time of epidemic.

An epidemic of small-pox is overdue. Efforts have been often made to show that infective diseases, and especially small-pox, recur in epidemic form with more or less definite periodicity. A more reasonable assumption is that of Hirsch, that the two factors essential for an outbreak of small-pox are the susceptibility of a number of persons to the morbid poison and the introduction of the virus. The present period of freedom, since the epidemic of 1901-2, has been an unusually prolonged one.

Inherent to the rarity of small-pox during recent years are dangers, other than those already mentioned, due to growing unfamiliarity with a disease which has long been known to show often a marked tendency to variation apart from the modification resulting from vaccination. Cases having been rare, and having been promptly removed to hospital, treatment has been carried out by specialists. In populous centres the general medical practitioner has frequently been relieved even of the responsibility of diagnosis. Diagnosis has, at the same time, become the more difficult because small-pox of the type made classic by text-book description has become increasingly rare. The accepted basis of differentiation between small-pox and varicella remains, but small-pox in the vaccinated presents modifications extremely misleading to those unfamiliar with them. Instances are not unknown of concurrent epidemics of chicken-pox and modified small-pox arising in schools and causing much difficulty and confusion. Even more puzzling and dangerous are those cases of malignant small-pox which are rapidly fatal from profound toxæmia, and in which the specific rash may be absent or may be replaced by purpuric or toxic rashes of aberrant type. Such cases, especially if occurring early in an epidemic, may easily be missed: a wrong diagnosis has been known to lead to disastrous consequences.

Of very great importance and interest are some epidemics of small-pox of very mild type which have occurred in recent years. Sydney, New South Wales, suffered from such an epidemic in 1913.* The earlier cases were exceptionally mild. Unusually severe chicken-pox was at the time prevalent, and even such of the patients as sought medical advice were at first believed to be suffering from the latter complaint. Prophecies of the dire results which must follow upon the introduction of small-pox amongst a community which had neglected infantile vaccination were not fulfilled at Sydney: on the other hand, infantile vaccination alone did not suffice to prevent such widespread and fatal epidemics as those of Sheffield, Warrington, and Middlesbrough. The Sydney outbreak was stamped out by modern methods of control, including the vaccination of contacts; and it is of interest to know that it "was almost entirely confined to the poorer neighbourhoods, because the better-educated classes invariably sought vaccination or re-vaccination as soon as the existence of small-pox in an epidemic form was publicly notified."

* *Proceedings of the Royal Society of Medicine.* Section of Epidemiology and State Medicine. Vol. VIII., No. 2. December 1914.

